# Dietary supplementation with a mixture of herbal extracts during late gestation and lactation improves performance of sows and nursing piglets through regulation of maternal metabolism and transmission of antibodies

**DOI:** 10.3389/fvets.2022.1026088

**Published:** 2022-09-23

**Authors:** Li Wang, Bin Huo, Lingjie Huang, Lianqiang Che, Bin Feng, Yan Lin, Shengyu Xu, De Wu, Zhengfeng Fang

**Affiliations:** ^1^Key Laboratory for Animal Disease Resistance Nutrition of the Ministry of Education, Institute of Animal Nutrition, Sichuan Agricultural University, Chengdu, China; ^2^Sichuan Dekon Livestock Foodstuff Group, Chengdu, China; ^3^College of Food Science, Sichuan Agricultural University, Ya'an, China

**Keywords:** *scutellaria baicalensis*, *lonicera japonica*, sows, performance, immunity

## Abstract

The dietary inclusion of phytogenic feed additives to improve the performance and health of sows is considered to be safe, effective and environmentally friendly, thus gaining growing popularity among new strategies. This study was designed with three trials aimed to determine the effective supplemental levels of *Scutellaria baicalensis* and *Lonicera japonica* mixed extracts (SLE) in sow diets based on production performance and explore its related mechanisms of action based on serum metabolites, antioxidant capacity, and immune profile of sows and nursing piglets. Trials 1 and 2 were conducted to determine the effective dose and ratio of SLE by supplementation of various proportions and doses of SLE to sows diets from the late pregnancy to weaning, with litter performance at farrowing and weaning and disease conditions being evaluated. Trial 3 was conducted to further explore the mechanisms of action of SLE as evaluated by serum immunity and antioxidants indices in late gestation and lactation sows. The results of trials 1 and 2 showed that dietary supplementation of 1.0 g/kg SLE (50% *S. baicalensis* extract, 30% *L. japonica* extract, and 20% wheat bran fiber as carrier) enhanced the number of piglets born alive, litter birth weight, litter weight gain, and average daily feed intake of sows during lactation, while decreased diarrhea of suckling piglets. In Trial 3, compared with the control group, dietary SLE supplementation increased (*P* < 0.05) sow serum glucose (GLU), triglyceride (TG), total cholesterol (TC), prolactin (PRL) and interleukin-10 (IL-10) concentrations, and total superoxide dismutase (T-SOD) activities at the farrowing, and increased (*P* < 0.05) sow serum prolactin, leptin, and insulin concentrations at d 14 of lactation. Fat concentrations in sow colostrum and in milk on day 14 of lactation, both IgA and IgG concentrations in colostrum, and both IL-10 and IgA concentrations in piglet serum at d 14 of lactation were all increased (*P* < 0.05) following dietary SLE supplementation. Altogether, dietary supplementation with the appropriate levels of SLE promoted health and growth of suckling piglets, which was associated with the improvement of maternal metabolism and transmission of antibodies.

## Introduction

Milk yield is one of the most important factors limiting neonatal piglet growth (1). Poor milk yield is the most frequent cause of breastfeeding failure (2). For sow-reared piglets, maximum weight gain is limited to as early as seven days after farrowing (3, 4). Besides, genetic selection for highly prolific sows has increased nutrient requirements during gestation and lactation to allow an increased milk yield to support large piglets (4). However, the vigorous metabolism of high-yielding sows during lactation generated extensive reactive oxygen species (ROS) to cause oxidative stress in the body, which resulted in reduced feed intake and milk yield of sows. The growth rate of suckling piglets largely depends on sow milk yield, but the amount of milk produced by sows did not improve significantly, resulting in the slow growth of piglets and the increase of weak and dead piglets (5). The limited nutrient intake would leave sows under severe catabolic status and reduce reproductive performance concurrently (6). One way to increase sow milk yield would be to stimulate mammary development. But sows enter a critical period of mammary gland development and rapid fetal growth at 75 days later in gestation. Maternal metabolic intensity increases and ROS accumulate in the body, which results in increased maternal oxidative stress and immunosuppression, and increased stillbirth and postpartum inflammation. In addition, maternal oxidative stress and low immune status will reduce the content of immune factors in colostrum and change the milk composition, increase the risk of diarrhea in piglets, and cause poor growth and development and even death of piglets. Therefore, optimizing feed intake during lactation, slowing down progressive oxidative stress, and improving immune function are the keys to improving the reproductive performance of sows. It is becoming more and more urgent to find an additive that can improve the milking ability and immune function of sows.

Several herbs and spices are assumed to have beneficial effects on milk secretion. Many herbal products are currently used in the European Union and elsewhere by the feed industry as feed additives (7). Traditional Chinese medicine (TCM), after a long period for being screened, is considered natural, low/non-toxic, showing short resistance and less residue. And its extracts have more functions including antibacterial and antioxidant activities, improving immunity, and regulating hormone secretion (8). *Scutellaria baicalensis* (*S. baicalensis*) Georgi and *Lonicera japonica* (*L. japonica*) Thunb. are two widely used traditional Chinese herbal medicines, and are officially listed in the Chinese Pharmacopeia. *S. baicalensis* roots have been used as anti-inflammatory and anticancer agent, for the treatment of bacterial and viral infections of the respiratory and the gastrointestinal tract, and because of its cholagogic, diuretic, and detoxifying properties. Baicalin is the most abundant component of *S. baicalensis* extracts, which could alleviate the adverse effect of heat stress and showed anti-allergic, anti-tumor, anti-inflammatory, and antioxidant activities (9, 10). *Lonicera japonica* contains a variety of organic acids, essential oils, flavones, saponins, and iridoids. Among them, chlorogenic acid and essential oils are the primary pharmacological compounds in *L. japonica*. Modern pharmacological studies showed that *L. japonica* and its extracts possessed wide pharmacological actions, such as antibacterial, anti-inflammatory, antiviral, antiendotoxin, antioxidant, antipyretic, and excitation of nerve centers (11–13). In clinical practice, the *L. japonica* extracts have been used for the treatment of fever, heatstroke, bloody flux, sores, carbuncles, furunculosis, headache, and some infectious diseases (11).

In weaning and growing-finishing pigs, many positive reports are available on the herbal extract supplementation diets (14–17). However, there is little information on responses of gestation and lactation sows to dietary herbal extract supplementation. Therefore, the objectives of the present study were to determine the effect of dietary herbal supplements on performance of sows and their progeny, and explore the underlying mechanisms as evaluated by serum metabolites, antioxidant capacity, and immune function of sows and suckling piglets.

## Materials and methods

### Ethics approval

The protocol of this study was approved by the Animal Care and Use Committee of Animal Nutrition Institute, Sichuan Agricultural University (Ethics Approval Code: SCAUAC201606-6), and was carried out in accordance with the National Research Council's Guide for the Care and Use of Laboratory Animals.

### Animals, diets, and experimental design

The study was designed with three trials. Trials 1 and 2 were conducted to determine the effective dose and ratio of *S. baicalensis* (SBE) and *L. japonica* (LJE) mixed extracts (SLE) by supplementation of various proportions and doses of SLE to sow diets from the late pregnancy to weaning, with litter performance at farrowing and weaning and disease conditions being evaluated. Trial 3 was conducted to further explore the mechanisms of action of SLE as evaluated by immunity and antioxidants indices during the late gestation and lactation period. Basal diets for all sows contained corn as a cardinal energy source and soybean meal as a cardinal protein source. Diets for sows during the late pregnancy (from day 85 of gestation to farrowing) and lactation were formulated to meet or exceed the NRC (2012) recommendations (Table 1). The herbal extract from *S. baicalensis* and *L. japonica* was extracted by a combination of ultrasound and microwave systems. The SLE products were provided by Beijing Centre Biology Co., Ltd., (Beijing, China).

**Table 1:** Composition and nutrient levels of the basal diets (air-dry basis).

**Items**	**Content**
**Ingredients, %**
Corn, 7.8% CP	42.11
Soybean meal, 46% CP	16.00
Barley, bark	15.0
Wheat bran	10.0
Puffed soybean, wet	8.0
Soybean oil	1.85
Fish steak powder	1.5
Calcium bicarbonate	1.27
Saccharose	1.0
Limestone	0.8
NaCl	0.4
L-Lysine sulfate, 70%	0.45
Vitamin premix^a^	0.50
Mineral premix^b^	0.30
Other	0.82
Total	100
**Nutrient levels**
DE, MJ/Kg	13.70
CP, %	17.98
SID-Lysine	1.00
SID-Methionine	0.41
SID-Methionine+Cystine	0.64
SID-Threonine	0.75
SID-Tryptophan	0.2
SID-Valine	0.82
Ca, %	0.85
P, %	0.67

^a^Provided per kg of complete diet: vitamin A, 35,000 IU; vitamin D, 36,000 IU; vitamin E, 50 IU; vitamin K_3_, 1.8 mg; riboflavin, 11 mg; niacin, 8 mg; D-pantothenic acid, 75 mg; biotin, 1.5 mg; folic acid, 2 mg; choline, 160 mg; vitamin B_6_, 5 mg; and vitamin B_12_, 3 mg.

^b^Provided per kg of complete diet: Fe (as FeSO_4_·7H_2_O), 90 mg; Cu (as CuSO_4_·5H_2_O), 20 mg; Zn (as ZnSO_4_), 100 mg; Mn (as MnO_2_), 25 mg; I (as KI), 0.14 mg; and Se (as Na_2_SeO_3_·5H_2_O), 0.15 mg.

The objective of Trial 1 was to determine the optimal proportion of the two extracts (SBE and LJE) in the SLE mixture as evaluated by the farrowing and lactation performance. Trial 1 was conducted in a pig farm in Fujian province from July to August, with an average room temperature being 28–30°C. According to a completely randomized block design, a total of 75 Yorkshire × Landrace sows (weighing 275.55 ± 20.97 kg, mean parity 4.44 ± 1.84) on the 85^th^ day of pregnancy were assigned based on genetic background, parity and body weight to five experiment groups: control (CON, *n* = 15; basal diet, Table 1), treatment group 1 (TRT1, *n* = 15, basal diet + 1 g/kg SLE powder product consisting of 45% SBE, 35% LJE and 20% wheat bran as carrier); treatment group 2 (TRT2, *n* = 15, basal diet + 1 g/kg a mixture of 50% SBE, 30% LJE, and 20% carrier); treatment group 3 (TRT3, *n* = 15, basal diet + 1 g/kg a mixture of 55% SBE, 25% LJE, and 20% carrier); and treatment group 4 (TRT4, basal diet +1 g/kg a mixture of 60% SBE, 20% LJE, and 20% carrier).

The objective of Trial 2 was to determine the optimal dietary supplementation level of the SLE mixture (50% SBE, 30% LJE, and 20% carrier) that was observed to be effective in improving sow performance in Trial 1. Trial 2 was carried out on a pig farm in Sichuan province from November to December. A total of 75 Yorkshire × Landrace sows (weighing 261.42 ± 25.74 kg, mean parity 3.13 ± 1.87) on the 85th day of pregnancy were assigned based on parity and body weight to five experiment groups: Control (CON, *n* = 15; basal diet, Table 1), 0.6TRT2 (basal diet + 0.6 g/kg SLE), 0.8TRT2 (basal diet + 0.8 g/kg SLE), 1.0TRT2 (basal diet + 1.0 g/kg SLE), and 1.2TRT2 (basal diet + 1.2 g/kg SLE). The basal diet was isoenergy but 1.5% lower in CP than diet shown in Table 1.

The objective of Trial 3 was to further verify the positive role of dietary SLE mixture supplementation at 1.0 g/kg diet (Table 1) on farrowing and lactation performance, and at the same time explore the mechanisms of action of SLE as evaluated by immunity and antioxidants indices based on blood and milk samples. Trial 3 was carried out on a pig farm in Sichuan province from May to July. A total of 64 Yorkshire × Landrace sows (mean weight 250.15 ± 10.35 kg, mean parity 2.70 ± 1.43) on the 85^th^ day of pregnancy were assigned based on genetic background, parity and body weight to two experiment groups: Control (CON, *n* = 32; basal diet) and 1.0 TRT2 (SLE, *n* = 32, basal diet + 1 g/kg of SLE).

### Feeding management

Sows were fed 3.0 kg/d from d 85 to 112 of pregnancy, and then fed 2.0 kg/d from d 113 of pregnancy to farrowing. After farrowing, sows were fed 2.0 kg of diet, which was increased by 1 kg/d in the following 3 days, and then sows had free access to feed from d 5 of lactation until weaning. During the gestation period, sows were housed in individual cages with concrete floors and equipped with automated drop feeders and nipple drinkers. On d 109 of gestation, sows were transported to the farrowing facility, where they were housed in individual farrowing crates (2.4 × 2.2 m) with creep area and nipple drinkers. Litters were standardized to be 12–14 piglets within 48 h after birth. Voluntary feed intake was measured daily throughout the lactation period. According to the practical situation of the pig farm in each trial, piglets were weaned at 21, 20, and 17 d postnatal, respectively, in trials 1, 2, and 3.

The feed consumption during lactation was recorded for each sow to calculate the average daily feed intake (ADFI). During the experimental period, numbers of piglets alive and dead per litter were recorded to calculate the survival ratio. Piglet body weight (BW) was recorded on d 1 (within 12 h of birth) and weaning day. In the 48th h after birth, the number of piglets in sows was adjusted so that litter piglets of sows were consistent among groups, and the number of piglets and litter weight were recorded. No creep feed was offered to piglets throughout the lactation period. The health status of sows and piglets were recorded daily during the lactation period. After weaning, the estrus interval (within 7 days) was recorded for each sow.

### Sample collection

In Trial 3, blood samples of sows were collected from the ear vein using sterile vacuum tubes from 20 randomly chosen sows on d 90 of gestation and d 1, d 14 of lactation before the morning feeding. At d 14 of lactation, blood samples were taken by jugular venipuncture using sterile vacuum tubes from 40 piglets selected from the 20 sows used for sow blood collection, with one male and one female piglet selected from each sow. After blood collection, serum were separated by centrifugation at 3,000 rpm for 5 min and then serum samples were collected and stored in sterile tubes at −20°C until analysis.

Colostrum was collected from 20 randomly chosen sows on d 1 of lactation within 4 h after initiation of farrowing. Milk was collected on d 14 of lactation after intramuscular injection of 10 IU of oxytocin behind the ear. After collection, milk (5 ml) for determination of hormone and immune indices was centrifuged (3,500 rpm) for 20 min to remove the fats and then the supernatant was collected and stored at −20°C until analysis.

### Chemical analysis

The glucose (Glu), triglyceride (TG), total cholesterol (TC), and free fatty acid (FFA) were measured using an auto-analyzer (BMD/Hitachi 7050 Auto Analyser; Japan). Whole milk was analyzed for dry matter, protein, fat, and lactose contents by a Milkyway-cp2 rapid milk composition analyzer (Institute of Food Science and Fermentation Engineering, Zhejiang University). The cytokines (IL-8, IL-10, TNF-α) and immunoglobulin (IgG, IgA, IgM) concentrations in colostrum, milk, and serum samples were determined using specific pig-ELISA quantification kits (Nanjing Jiancheng Bioengineering Institute, Nanjing, China) according to instructions of their respective manufacturers, respectively. The serum concentrations of insulin (INS), prolactin (PRL), thyroid-stimulating hormone (TSH), leptin, and growth hormone (GH) in sows were measured by respective ELISA quantification kits (Nanjing Jiancheng Bioengineering Institute, Nanjing, China). Assessment of total antioxidant capacity (T-AOC) and total superoxide dismutase (T-SOD) in serum from sows and piglets were performed using specific assay kits (Nanjing Jiancheng Bioengineering Institute, Nanjing, China).

### Statistical analyses

All data were checked for normality using the univariate procedure of Statistical Analysis Software (Version 9.4, SAS Institute Inc., Cary, NC, USA) and transformed, if required. The data of litter weight at weaning, litter weight gain and the number of weaned piglets were assessed by analysis of covariance using the general linear model (GLM) procedure of SAS, and the adjusted litter weight and litter number were used as covariates. The data of piglets born alive, litter birth weight, and piglet birth weight were assessed using the total number born as covariate by covariance analysis. The data of diarrhea of piglets and estrous rate were analyzed by χ^2^-test. The data of the estrus interval was analyzed by rank-sum test. The other data were analyzed with one-way analysis of variance (ANOVA) using the GLM procedure of SAS software. CONTRAST was used to compare differences with the control group. Differences were considered significant when *P* ≤ 0.05, and trends were noted when 0.05 < *P* < 0.10. Quadratic effects were not significant, and hence their *P*-values were omitted from the tables.

## Results

### Sows and piglets performance

As presented in Table 2, the performance of sows and piglets was affected by diet. Sows in the TRT2 showed significantly increased total number born, number born alive, litter birth weight, litter weight and piglet weight of weaning at 21 days, litter weight gain, total intake and ADFI of sows at day 21 of lactation, and significantly decreased piglets diarrhea compared with sows in the CON treatment (*P* < 0.05). However, the number of weaned piglets, piglet birth weight, weaning-estrous interval, and estrous rate within 7 days were not affected (*P* > 0.05) by SLE dietary supplementation. TRT3 and TRT2 had the same trend, but no difference (*P* > 0.05) in reproductive performance was observed between other treatments. Therefore, we would choose TRT2 [50% SBE, 30% LJE, and 20% carrier (wheat bran), SLE] as the optimized mixed proportion for Trial 2.

**Table 2:** Reproductive performance of sows (Trial 1).

	**Treatments** ^**1**^					
**Items**	**CON**	**TRT1**	**TRT2**	**TRT3**	**TRT4**	**SEM^2^**
Average parity	4.5	4.3	4.5	4.6	4.2	2.56
**Litter, no**
Total number born	12.1^b^	12.9^ab^	14.4^a^	13.6^a^	11.7^b^	1.88
Piglets born alive	11.5^b^	12.4^ab^	13.9^a^	13.1^a^	11.6^b^	1.80
Piglets adjusted^3^	11.1	11.0	11.3	11.4	10.9	0.55
Piglets weaned	10.6	10.7	10.9	10.3	10.4	0.66
**BW, kg**
Litter birth weight	15.36^b^	17.68^ab^	19.00^a^	16.99^ab^	17.20^ab^	2.12
Piglet birth weight	1.36	1.47	1.39	1.34	1.51	0.13
**Weaning, 21 days**
Litter weight after adjusted^3^	18.33	17.22	18.85	18.63	18.80	2.00
Litter weight	55.36^b^	59.16^ab^	65.18^a^	62.02^a^	58.71^ab^	5.37
Piglet weight	5.35^b^	5.53^ab^	6.02^a^	6.03^a^	5.67^ab^	0.36
Litter weight gain	37.54^b^	41.67^ab^	46.94^a^	45.97^a^	40.79^ab^	4.18
**Feed intake, kg, 21 days**
Total intake	90.68^c^	96.33^bc^	99.99^ab^	103.41^a^	99.37^ab^	4.73
ADFI	4.32^c^	4.59^bc^	4.76^ab^	4.92^a^	4.73^ab^	0.14
**Post-weaning estrus, within 7 days**
Estrus interval, d	5.89	5.31	5.58	5.67	5.50	0.24
Estrous rate, %	69.23%	86.67%	86.67%	85.71%	69.23%	0.41
Diarrhea of piglets, %	33.62%^a^	17.68%^b^	12.34%^b^	12.37%^b^	12.03%^a^	0.04

^1^CON, n = 15; basal diet, TRT1, n = 15, control+ 1 g/kg a mixture of 45% SBE, 35% LJE, and 20% carrier (wheat bran); TRT2, n = 15, control+1 g/kg a mixture of 50% SBE, 30% LJE, and 20% carrier (wheat bran); TRT3, n = 15, control+1 g/kg a mixture of 55% SBE, 25% LJE, and 20% carrier (wheat bran); TRT4, n = 15, control+1 g/kg a mixture of 60% SBE, 20% LJE, and 20% carrier (wheat bran).

^2^Standard error of the means.

^3^In the 48th h after birth, adjusted the number of piglets and litter weight.

^a, b^Means within a row without a common superscript differ, P < 0.05.

As presented in Table 3, compared with sows in the CON treatment, sows in the 1.0TRT2 significantly increase the total number born, number born alive, number of weaned piglets, litter birth weight, litter weight and piglet weight of weaning at 20 days, litter weight gain, total intake and ADFI of sows at day 20 of lactation (*P* < 0.05) and significantly decreased piglets' diarrhea (*P* < 0.05). There was no effect (*P* > 0.05) on piglet weight of birth, weaning-estrous interval, and estrous rate. In addition, 0.6TRT2 sows had higher total feed intake and ADFI (20 days) (*P* < 0.05) compared with CON sows. No difference (*P* > 0.05) in reproductive performance was observed among treatments. Therefore, the 1.0TRT2 (1.0 g/kg SLE) was selected as the optimum content for Trial 3.

**Table 3:** Reproductive performance of sows (Trial 2).

	**Treatments** ^**1**^				
**Items**	**CON**	**0.6TRT2**	**0.8TRT2**	**1.0TRT3**	**1.2TRT4**	**SEM^2^**
Average parity	3.07	3.07	3.13	3.27	3.13	1.12
**Litter, no**
Total number born	10.5^b^	11.4^ab^	11.2^ab^	11.9^a^	12.5^a^	1.57
Piglets born alive	10.1^b^	10.5^ab^	10.7^ab^	11.6^a^	12.4^a^	2.16
Piglets adjusted^3^	11.1	11.1	11.1	11.3	11.5	0.56
Piglets weaned	10.1^b^	10.5^ab^	10.7^ab^	11.1^a^	10.9^ab^	0.63
**BW, kg**
Litter birth weight	14.53^b^	15.35^ab^	15.23^ab^	17.00^a^	16.64^a^	2.24
Piglet birth weight	1.45	1.52	1.48	1.48	1.37	0.16
**Weaning, 20 days**
Litter weight after adjusted^3^	16.00	16.17	16.92	16.17	16.01	1.63
Litter weight	57.46^b^	62.43^b^	63.76^b^	71.02^a^	63.19^b^	5.63
Piglet weight	5.71^b^	5.99^b^	6.03^b^	6.43^a^	5.82^b^	0.52
Litter weight gain	43.58^b^	47.34^ab^	48.73^ab^	54.99^a^	48.29^ab^	4.70
**Feed intake, kg, 20 days**
Total intake	91.81^b^	109.93^a^	102.58^ab^	107.28^a^	101.01^ab^	8.91
ADFI	4.59^b^	5.50^a^	5.13^ab^	5.36^a^	5.05^ab^	0.45
**Post-weaning estrus, within 7 days**
Estrus interval, d	5.42	4.91	4.77	5.15	5.07	0.60
Estrous rate, %	85.71%	78.57%	93.33%	86.67%	93.33%	0.38
Diarrhea of piglets, %	22.58%^a^	10.32%^b^	7.78%^b^	3.47%^b^	8.23%^b^	0.10

^1^CON, n = 15, basal diet; 0.6TRT2, n = 15, control+0.6 g/kg SLE; 0.8TRT2, n = 15, control+0.8 g/kg SLE; 1.0TRT2, n = 15, control+1.0 g/kg SLE; 1.2TRT2, n = 15, control+1.2 g/kg SLE.

^2^Standard error of the means.

^3^In the 48th h after birth, adjusted the number of piglets and litter weight.

^a, b^Means within a row without a common superscript differ, P < 0.05.

As presented in Table 4, the lactation ADFI and total intake (17 days) were significantly increased (*P* < 0.01) by dietary supplementation of 1.0 g/kg SLE. In addition, compared with the CON group, the 1.0TRT2 group had significantly increased (*P* < 0.05) litter weight at weaning and litter weight gain and decreased (*P* < 0.05) diarrhea of piglets during lactation. The number of piglets born alive, litter birth weight, number of weaned piglets, and weaning-estrous interval were not affected (*P* > 0.05) by SLE supplementation.

**Table 4:** Reproductive performance of sows (Trial 3).

	**Treatments** ^**1**^		
**Items**	**CON**	**1.0TRT2**	**SEM^2^**
Average parity	2.75	2.66	0.36
**Litter, no**
Total number born	14.26	14.97	0.78
Piglets born alive	13.39	13.63	0.71
Piglets adjusted^3^	12.96	12.70	0.33
Piglets weaned	12.0	12.30	0.41
**BW, kg**
Litter birth weight	18.53	19.01	0.95
Piglet birth weight	1.39	1.42	0.05
**Weaning, 17 days**
Litter weight after adjusted^3^	18.68	18.59	0.87
Litter weight	53.72^b^	59.05^a^	2.54
Piglet weight	4.47	4.79	0.31
Litter weight gain	35.10^b^	40.16^a^	2.58
**Feed intake, kg, 17 days**
Total intake	70.06^B^	79.32^A^	14.68
ADFI	4.16^B^	4.67^A^	0.27
**Post-weaning estrus, within 7 days**
Estrus interval, d	4.2	4.5	0.24
Estrous rate, %	93.33%	92.31%	0.36
Diarrhea of piglets, %	9.04%^A^	1.24%^B^	0.05

^1^CON, n = 32, basal diet; 1.0TRT2, n = 32, control+1.0 g/kg SLE.

^2^Standard error of the means.

^3^In the 48th h after birth, adjusted the number of piglets and litter weight.

^A, B, a, b^The values with unlike superscripts differ at P < 0.05 (small letters) or P < 0.01 (capital letters).

### Blood profiles of sows

As presented in Table 5, there were no differences (*P* > 0.05) in serum concentrations of GLU, TG, TC, and FFA between groups at d 90 of gestation and d 14 of lactation. Compared with the CON group, dietary SLE supplementation significantly increased serum GLU (*P* < 0.01) and TG and TC (*P* < 0.05) concentrations at the farrowing day (Table 5).

**Table 5:** Serum biochemical indices of sows (Trial 3), mmol/L.

	**Treatments** ^**1**^		
**Items**	**Con**	**1.0TRT2**	**SEM^2^**
**GLU**
d 90 of gestation	4.64	4.61	0.42
d 1 of lactation	3.77^B^	4.22^A^	0.16
d 14 of lactation	3.82	4.26	0.35
**TG**
d 90 of gestation	0.39	0.55	0.08
d 1 of lactation	0.70^b^	0.90^a^	0.10
d 14 of lactation	0.41	0.42	0.09
**TC**
d 90 of gestation	1.57	1.56	0.12
d 1of lactation	1.71^b^	1.93^a^	0.09
d 14 of lactation	2.08	2.18	0.16
**FFA**
d 90 of gestation	0.81	0.85	0.13
d 1of lactation	0.03	0.04	0.01
d 14 of lactation	0.44	0.57	0.15

^1^CON, n = 10, basal diet; SLE, n = 10, control+1.0 g/kg SLE.

^2^Standard error of the means.

^A, B, a, b^The values with unlike superscripts differ at P < 0.05 (small letters) or P < 0.01 (capital letters).

### Serum hormones of sows

As presented in Table 6, there were no differences (*P* > 0.05) in the concentrations of GH, TSH, PRL, INS, and leptin in sow serum between groups at d 90 of gestation. Compared with the CON group, dietary SLE supplementation significantly increased sow serum PRL concentrations at the farrowing day and significantly increased serum PRL, leptin, and INS concentrations at d 14 of lactation (*P* < 0.05). However, the concentrations of GH and TSH in sow serum on days 1 and 14 of lactation were not affected (*P* > 0.05) by SLE diet treatments.

**Table 6:** Serum hormones of sows (Trial 3).

	**Treatments** ^**1**^		
**Items**	**Con**	**1.0TRT2**	**SEM^2^**
**GH, ng/ml**
d 90 of gestation	6.35	6.62	1.34
d 1 of lactation	4.90	4.57	0.96
d 14 of lactation	3.99	4.60	0.65
**TSH, mIU/L**
d 90 of gestation	7.00	7.18	1.99
d 1 of lactation	3.31	3.95	0.77
d 14 of lactation	2.46	4.20	1.41
**PRL, ng/ml**
d 90 of gestation	2.52	2.61	1.10
d 1 of lactation	22.24^b^	32.00^a^	4.69
d 14 of lactation	5.25^b^	8.63^a^	1.40
**Leptin, ng/ml**
d 90 of gestation	11.46	11.68	1.71
d 1 of lactation	10.53	12.77	1.39
d 14 of lactation	8.85^b^	11.17^a^	0.56
**INS, mIU/L**
d 90 of gestation	63.86	64.38	3.50
d 1 of lactation	75.71	81.85	5.39
d 14 of lactation	40.99^b^	54.37^a^	4.45

^1^CON, n = 10, basal diet; SLE, n = 10, control+1.0 g/kg SLE.

^2^Standard error of the means.

^a, b^The values with unlike superscripts differ at P < 0.05 (small letters) or P < 0.01 (capital letters).

### Serum immunization index of sows

As presented in Table 7, compared with the CON group, dietary SLE supplementation significantly increased serum IL-10 (*P* < 0.05) concentrations at farrowing (Table 7). But the humoral immunity factor contents of IgA, IgG, IgM, IL-8, and TNF-α in sow serum were not affected (*P* > 0.05) by dietary SLE supplementation.

**Table 7:** Serum immunization index of sows (Trial 3).

	**Treatments** ^**1**^		
**Items**	**Con**	**1.0TRT2**	**SEM^2^**
**IgA, mg/ml**
d 1 of lactation	0.90	0.93	0.25
d 14 of lactation	1.28	1.34	0.35
**IgG, mg/ml**
d 1 of lactation	10.99	13.31	2.45
d 14 of lactation	12.79	19.23	5.02
**IgM, mg/ml**
d 1 of lactation	3.64	3.44	0.57
d 14 of lactation	4.95	5.17	1.19
**IL-10, ng/L**
d 1 of lactation	49.98^b^	59.60^a^	4.48
d 14 of lactation	63.43	68.59	6.61
**IL-8, ng/L**
d 1 of lactation	146.09	150.32	39.20
d 14 of lactation	190.73	183.13	46.06
**TNF-a, ng/L**
d 1 of lactation	110.55	117.17	11.2
d 14 of lactation	103.39	106.15	8.75

^1^CON, n = 10, basal diet; SLE, n = 10, control+1.0 g/kg SLE.

^2^Standard error of the means.

^a, b^The values with unlike superscripts differ at P < 0.05 (small letters) or P < 0.01 (capital letters).

### Colostrum and milk conventional ingredients

Colostrum and milk composition (i.e., dry matter, protein, and lactose) was not affected by dietary treatment (*P* > 0.05). Compared with the CON group, dietary SLE supplementation significantly increased fat concentrations in sow colostrum and milk on day 14 of lactation (*P* < 0.05; Table 8). As presented in Table 9, sows receiving SLE had significantly higher colostrum concentrations of IgA (*P* < 0.05) and IgG (*P* < 0.01) than the CON sows. However, no significant difference (*P* > 0.05) was observed in concentrations of IL-10, IgM, IL-8, and TNF-a in colostrum and milk on day 14 of lactation between the two groups.

**Table 8:** Colostrum and milk conventional ingredients of sows (Trial 3), %.

	**Treatments** ^**1**^		
**Items**	**Con**	**1.0TRT2**	**SEM^2^**
**Protein**
Colostrum	6.62	6.95	0.37
Milk of d 14	3.90	3.94	0.18
**Fat**
Colostrum	3.83^b^	4.79^a^	0.47
Milk of d 14	6.31^B^	7.99^A^	0.47
**Lactose**
Colostrum	5.86	6.14	0.42
Milk of d 14	10.33	10.88	0.53
**Dry matter**
Colostrum	18.53	18.10	1.25
Milk of d 14	10.42	10.44	0.48

^1^CON, n = 10, basal diet; SLE, n = 10, control+1.0 g/kg SLE.

^2^Standard error of the means.

^A, B, a, b^The values with unlike superscripts differ at P < 0.05 (small letters) or P < 0.01 (capital letters).

**Table 9:** Colostrum and milk immune factors of sows (Trial 3).

	**Treatments** ^**1**^		
**Items**	**Con**	**1.0TRT2**	**SEM^2^**
**IgA, mg/ml**
Colostrum	1.02^b^	1.92^a^	0.31
Milk of d 14	0.52	0.72	0.20
**IgG, mg/ml**
Colostrum	18.86^B^	28.90^A^	2.45
Milk of d 14	0.91	1.59	0.38
**IgM, mg/ml**
Colostrum	1.13	1.77	0.44
Milk of d 14	0.57	0.76	0.20
**IL-10, ng/L**
Colostrum	20.51	22.10	5.70
Milk of d 14	9.88	12.39	1.91
**IL-8, ng/L**
Colostrum	136.51	142.28	16.52
Milk of d 14	63.52	59.97	10.11
**TNF-a, ng/L**
Colostrum	66.62	71.16	8.13
Milk of d 14	20.04	19.79	2.69

^1^CON, n = 10, basal diet; SLE, n = 10, control+1.0 g/kg SLE.

^2^Standard error of the means.

^A, B, a, b^The values with unlike superscripts differ at P < 0.05 (small letters) or P < 0.01 (capital letters).

### Serum immunization index of piglets

As presented in Table 10, compared with the CON group, dietary SLE supplementation significantly increased concentrations of IL-10 (*P* < 0.05) and IgA (*P* < 0.01) in serum at d 14 of piglets. There were no differences (*P* > 0.05) in concentrations of IgG, IgM, IL-8, and TNF-α in serum between groups at d 14 of piglets.

**Table 10:** Serum immune factors of piglets (Trial 3).

	**Treatments** ^**1**^		
**Items**	**Con**	**1.0TRT2**	**SEM^2^**
IgA, mg/ml	0.58^B^	1.02^A^	0.09
IgG, mg/ml	3.00	3.65	0.43
IgM, mg/ml	2.16	2.04	0.48
IL-10, ng/L	30.25^b^	42.73^a^	5.16
IL-8, ng/L	71.34	70.88	25.86
TNF-a, ng/L	80.78	84.85	3.76

^1^CON, n = 20, basal diet; SLE, n = 20, control+1.0 g/kg SLE.

^2^Standard error of the means.

^A, B, a, b^The values with unlike superscripts differ at P < 0.05 (small letters) or P < 0.01 (capital letters).

### Serum SOD and T-AOC activity levels of sows and piglets

**Figure 1:**
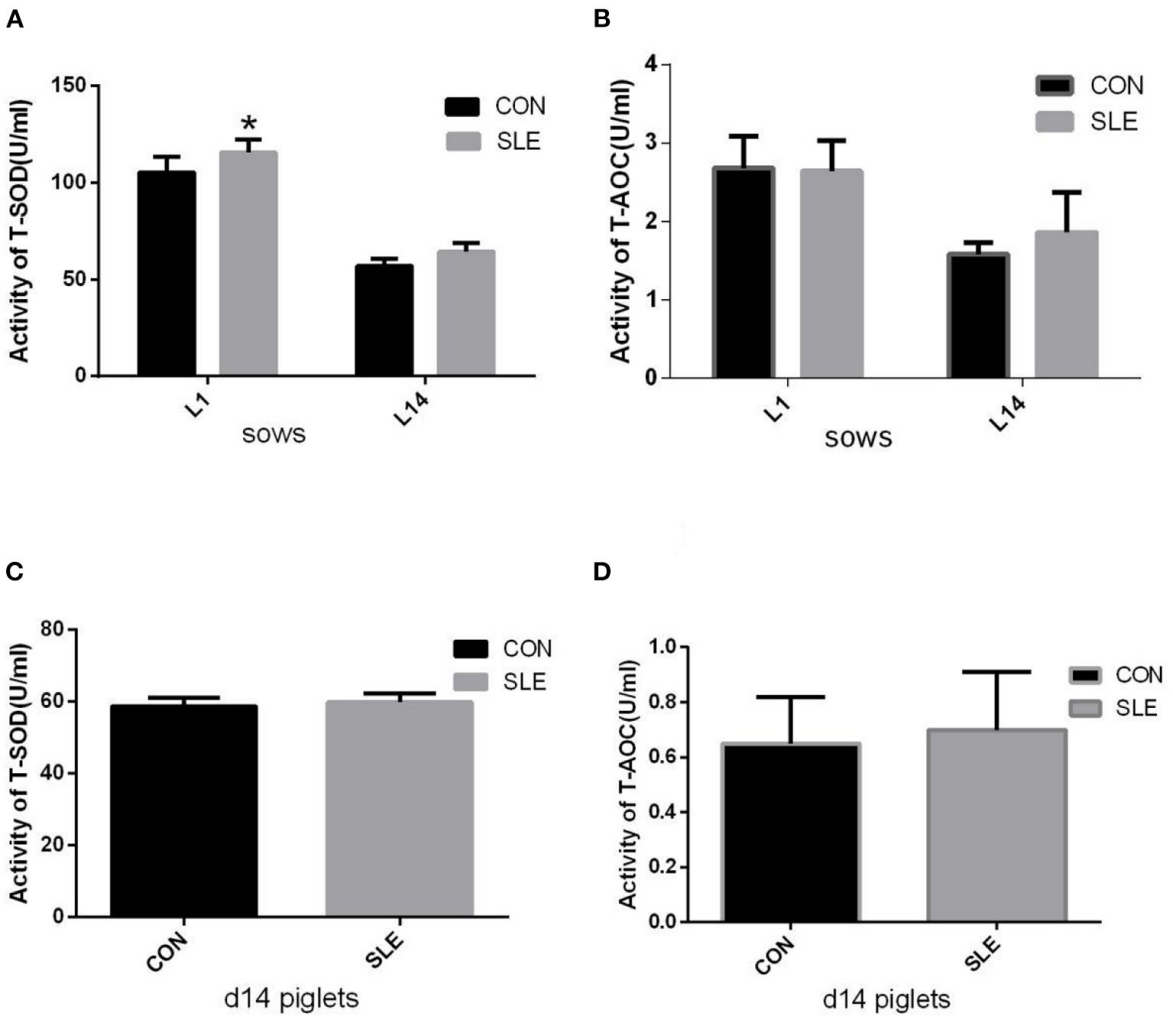
The effects of dietary *S. baicalensis* and *L. japonica* extract supplementation on serum anti-oxidative capacity of sows and piglets. **(A,B)** Total superoxide dismutase (T-SOD) and total antioxidant capacity (T-AOC) activities in serum of sows (*n* = 10); **(C,D)** T-SOD and T-AOC activities in serum of d14 piglets (*n* = 20). *Indicating a significant difference (*P* < 0.05). Data show the means ± standard deviation (SD).

The impact of dietary SLE supplementation on serum antioxidant indexes is shown in Figure 1. The T-SOD activity in sow serum on d 1 of lactation were significantly increased compared with the CON group (*P* < 0.05; Figure 1A). However, there were no differences (*P* > 0.05) in sow serum T-AOC activity between groups at farrowing and d 14 of lactation (Figure 1B). The activities of T-AOC and T-SOD in piglet serum were not affected by dietary SLE supplementation (Figures 1C,D).

## Discussion

Herbal medicines have been tested extensively in swine diets as potential alternatives to antibiotics growth promoters owing to antiviral, antibacterial, and antioxidant properties, stimulation of the immune system, and improvement of digestibility and absorption of nutrients (18–20). However, the clinical efficacy has huge differences with different compatibility ratios and dosages of herbs and extracts (21). According to the “*Chinese Pharmacopeia*” records, the proportion of *L. japonica* (chlorogenic acid 12 g/tube) and *S. baicalensis* (baicalin 24 g/tube) is 1:2 in the *Yinhuang Oral Liquid*, which is beneficial for fever, cough, hemoptysis, jaundice, dysentery, acute conjunctivitis, carbuncle, and furuncle, and prevents abnormal fetal movements (22). But despite these advantages, information available in the previous literature on the response of supplementing *L. japonica* and *S. baicalensis* in swine is scarce. In the present studies, dietary herbs extract mixture supplementation exerting positive effects on production performance in sows and nursing piglets were observed in all three trials, and the effective dose of SLE was observed to be 1.0 g/kg diet with the ratio of SBE and LJE in the SLE mixture being 50 and 30%, respectively. The feed intake during lactation period is a key factor to limit the production performance of sows, and maximum sow milk output requires that feed intake was maximized (4). Previous studies have confirmed that dietary herb extract supplementation could contribute to the desired organoleptic qualities of the diets and stimulate the appetite, as well as improve digestive tract function by increasing hydrochloric acid and enzyme secretion, thus improving the feed intake and lactation yield of sows (8, 23). Likewise, there were many reports on improved feed intake through herb extract additives in sows or pigs diets (15–18, 24–27). In addition, the herbal ingredients' metabolites can improve growth performance and reduce diarrhea in nursing piglets through breast milk (18, 28). Liu et al. indicated that dietary supplementation of herbal extract mixture (55% *S. baicalensis* and 25% *L. japonica*) at 0.5 and 1.0 g/d could alleviate heat stress, improve the feed intake and dry matter digestibility of sows during lactation, and reduce diarrhea and enhance daily gain in piglets (29), which were similar to observations in the current studies. However, the differences in the total number born may not be related to the SLE in this study because the SLE mixture added to the sow feed was started on the 85^th^ day of pregnancy.

A high feed intake of lactating sows can increase piglets' growth performance and positively influence subsequent reproduction (30). Furthermore, Oliviero et al. demonstrated that a sow diet has a profound effect on the robustness of piglets (31). In the current studies, piglets weaned from SLE-supplemented sows had a greater weaning weight and overall ADG than the CON group, which suggested that SLE supplementation improved sow milk production. This improvement in milk output may be due to enhanced feed intake and nutrient digestibility. Similarly, the inclusion of herbal extract blends in lactation diet were shown to enhance piglet performance and result in higher weight at weaning (32). Zhong et al. also observed that supplementation of 0.04% phytogenic additive to sows positively affected feed intake and milk production of sows and litter performance (33). In addition, Liu et al. demonstrated that the antibacterial activity and anti-inflammatory properties of *S. baicalensis* and *L. japonica* provided a beneficial effect on the immune system and even reduced subclinical or clinical infections, subsequently benefiting the health of the pigs, which could account for enhanced growth performance (18). Watson also confirmed that the inclusion of herbs could lead to an improvement in antibody levels of colostrum and increase milk quality (34). Redoy et al. suggested that dietary supplementation with herbs (*Plantago lanceolata L*. and *Allium sativum*) could prevent the undesirable microorganisms' reproduction and stimulate the secretion of antibodies, thus positively influencing the colostral concentration of IgA and IgG, serum immunocompetence, and growth performance of animals (35). On the other hand, the beneficial properties of herbal extract such as antimicrobial, antiviral, and stimulation of the immune system could improve uterine involution and protect the sow from possible postpartum urogenital infections (36). As expected, the current study indicated that SLE supplementation decreased piglets' diarrhea, which also could account for the enhanced growth performance in this study.

Notably, relative to control sows, sows fed SLE had increased serum concentrations of triglycerides, glucose, and cholesterol on farrowing. Modern genotype sows' farrowing is a long process, and yet prolonged labor could lead to dystocia and even stillbirth. And part of the reason for the prolonged labor is insufficient energy supply (18). Glucose in serum is the main source of energy for tissue cells in the sow. In this regard, improving serum concentrations of glucose and triglycerides could help to reduce sows' stress in farrowing, which is conducive to the progress of farrowing and reduces labor. The increased triglycerides and glucose concentrations in the serum of sows receiving herbal extracts indicate the latter's homeostasis-promoting effects (18, 37). Meng et al. and Ruan et al. also indicated that chlorogenic acid could regulate glucose metabolism, and improve lipids and enzymes involved in lipid metabolism in the organism (38, 39). More than 95% of the fatty acids in the cream were in the form of triglycerides, and the ingredients needed for the synthesis of fat in the milk were derived from blood fats (1). The content of triglycerides in serum of farrowing sows was significantly increased, suggesting that more triglycerides in plasma may be absorbed by the body from the peripheral circulation of the blood and used for the synthesis of milk fat (1). However, Yan et al. suggested that the increased concentrations of serum cholesterol and triglycerides might be resulted from homeostasis and the promotion of intestinal lipid absorption, respectively (16, 27). Myer et al. indicated that the formation of fat deposits depended on the level of serum triglyceride, which is accompanied by increased triglyceride and cholesterol levels. The possible reason was the increase of fatty acid synthase and the outcome of combined action of hormone sensitive enzyme activity (40). However, contrary to the results of this experiment, chlorogenic acid and baicalein had a certain regulating effect on body fat metabolism. Chlorogenic acid seemed to be more potent for bodyweight reduction and regulation of lipid metabolism than caffeic acid (41, 42). Baicalein reduces body cholesterol content by inhibiting cholesterol acyltransferase activity and cholesterol absorption, which was also clarified in earlier studies (18, 43–45).

Interleukin 10 (IL-10) is a pleiotropic cytokine with an extensive spectrum of biological effects in immunoregulation and inflammation (46). As previously reported, chlorogenic acid had a certain regulation effect on organism immunity and adaptive immunity in the regulation of inflammation and immunity, which can regulate the number of white blood cells, the function of macrophages, expression of cytokines secretion, and immune cell activation factors (47, 48). Baicalin and baicalein not only have obvious anti-inflammatory and immunosuppressive effects but also can improve the functions of macrophages and NK cells (49, 50). In the present study, serum IL-10 levels in sows and piglets increased, showing an improvement in cellular and humoral immunity of offspring in response to SLE supplementation of sows during late gestation and nursing period.

Besides providing energy and nutrients for piglets, sow colostrum's most important function was to activate the immune system and equip piglets with specific and non-specific immunity protection functions (46, 51). Feeding lactating sows a diet supplemented with SLE increased colostrum IgG and IgA concentrations in sows and serum IgA concentrations in piglets, indicating that the active compounds in SLE were deposited in sow milk. Wang et al. reported dietary herbal extracts supplementation increased colostrum IgG and IgA concentrations (52). Thus, these findings substantiate that dietary supplementation of sows with SLE during late gestation and lactation could significantly improve serum IgA and IL-10 of piglets. These may be because active substances in SLE from colostrum and milk effectively strengthen the provision of resources for supporting cells and the immune system of piglets by regulating lipid and protein metabolism (12, 13, 38). These results indicate that dietary SLE may improve weaned piglets' immune function and resistance to pathogenic microorganisms infection and attenuate stress injury on the organism.

The antioxidant status of an organism is critical for maintaining animal health and can be affected by nutrients (53, 54). Due to the antioxidant properties contained in herbs, the use of herbs as additives is important for the antioxidant system and stress tolerance of animals. Previous studies have stated that *S. baicalensis* inhibited lipid peroxidation in rat liver homogenate (55). Su et al. also clarified the antioxidant effect of *L. japonica* extract in rats (56). Shang et al. demonstrated that SLE supplementation generated a decrease in serum cortisol that could be attributed to the anti-stress and sedative properties (11). Consistent with the antioxidant function, the current study stated that supplementation of SLE mixture in sow diets significantly increased T-SOD activities in sow serum on day one of lactation. Wang et al. and Huang et al. elucidated that the antioxidant roles of *S. baicalensis* have been traced to several of its flavones, which include wogonin, baicalin, baicalein, and the skullcap flavone (57, 58). Choi et al. clarified that the anti-oxidative activity of *L. japonica* was attributed to polyphenols, flavones, iridoids, and saponins, which exhibit various antioxidant properties (59). Taking into account that increased systemic oxidative stress is observed throughout lactation in sows and that high energy metabolic demands in the lactation process accelerate mitochondrial oxidative stress and reactive oxygen species production (60, 61), it is necessary to prevent oxidative stress by SLE supplementation during lactation.

## Conclusion

In summary, this study demonstrated that supplementation of 1.0 g/kg SLE [50% *S. baicalensis*, 30% *L. japonica* extract mixture, and 20% carrier (wheat bran)] in sow diet in late gestation and during lactation was the optimum content and optimized mixed-proportion that could improve immunity and production performance of sows and nursing piglets. The SLE supplementation increased immune molecules in sows' serum and milk, which is beneficial to piglet health and growth through a transmission effect.

## Data availability statement

The raw data supporting the conclusions of this article will be made available by the authors, without undue reservation.

## Ethics statement

The animal study was reviewed and approved by Animal Care and Use Committee of Animal Nutrition Institute, Sichuan Agricultural University.

## Author contributions

ZF and DW designed the study. LW and BH performed experiments. LW, BH, LH, LC, BF, YL, and SX performed data analysis. BH, LW, and ZF wrote the draft and revised the manuscript. All authors have read and approved the manuscript.

## Funding

This work was supported by Beijing Centre Biology Co. Ltd. The funder was not involved in the study design, collection, analysis, interpretation of data, the writing of this article, or the decision to submit it for publication.

## Conflict of interest

The authors declare that the research was conducted in the absence of any commercial or financial relationships that could be construed as a potential conflict of interest.

## Publisher's note

All claims expressed in this article are solely those of the authors and do not necessarily represent those of their affiliated organizations, or those of the publisher, the editors and the reviewers. Any product that may be evaluated in this article, or claim that may be made by its manufacturer, is not guaranteed or endorsed by the publisher.

## References

[1] Boyd RD Kensinger RS. Metabolic Precursors for Milk Synthesis. The Lactating Sow (1998). Available online at: https://www.researchgate.net/publication/283276946 (accessed January 1, 1998).

[2] Zuppa AA Sindico P Orchi C Carducci C Cardiello V Romagnoli C. Safety and efficacy of galactogogues: substances that induce, maintain and increase breast milk production. J Pharm Pharm Sci. (2010) 13:162–74. 10.18433/J3DS3R 20816003

[3] NRC. Nutrient Requirements of Swine, 11th ed. National Research Council, Academic Press, Washington, DC (2012).

[4] Ball RO Samuel RS Moehn S. Nutrient requirements of prolific sows. In: Proceedings of the Advances in Pork Production: Proceedings of the Banff Pork Seminar. Edmonton (2008). Available online at: https://www.researchgate.net/publication/285264007

[5] Miller YJ Collins AM Smits RJ Thomson PC Holyoake PK. Providing supplemental milk to piglets preweaning improves the growth but not survival of gilt progeny compared with sow progeny. J Anim Sci. (2012) 90:5078–85. 10.2527/jas.2011-4272 22829606

[6] Sulabo RC Tokach MD Dritz SS Goodband RD DeRouchey JM Nelssen JL. Effects of varying creep feeding duration on the proportion of pigs consuming creep feed and neonatal pig performance. J Anim Sci. (2010) 88:3154–62. 10.2527/jas.2009-2134 20495115

[7] Maenner K Vahjen W Simon O. Studies on the effects of essential-oil-based feed additives on performance, ileal nutrient digestibility, and selected bacterial groups in the gastrointestinal tract of piglets. J Anim Sci. (2011) 89:2106–12. 10.2527/jas.2010-2950 21357448

[8] Wenk C. Herbs and botanicals as feed additives in monogastric animals. Asian Austral J Anim Sci. (2003) 16:282–9. 10.5713/ajas.2003.282

[9] Liao HF Ye J Gao LL Liu YL. The main bioactive compounds of *Scutellaria baicalensis* Georgi. for alleviation of inflammatory cytokines: a comprehensive review. Biomed Pharmacother. (2021) 133:110917. 10.1016/j.biopha.2020.110917 33217688

[10] Li J Khan IA. The advanced bioactivity studies of scutellaria baicalensis georgi and its phenolic compounds. Acta Hortic. (2006) 720:157–70. 10.17660/ActaHortic.2006.720.15

[11] Shang XF Pan H Li MX Miao XL Ding H. *Lonicera japonica* Thunb: ethnopharmacology, phytochemistry and pharmacology of an important traditional Chinese medicine. J Ethnopharmacol. (2011) 138:1–21. 10.1016/j.jep.2011.08.016 21864666 PMC7127058

[12] Ma FT Shan Q Jin YH Gao D Li HY Chang MN. Effect of *Lonicera japonica* extract on lactation performance, antioxidant status, and endocrine and immune function in heat-stressed mid-lactation dairy cows. J Dairy Sci. (2020) 103:10074–82. 10.3168/jds.2020-18504 32896406

[13] Shinomiya K Omichi J Ohnishi R Ito H Yoshida T Kamei C. Effects of chlorogenic acid and its metabolites on the sleep-wakefulness cycle in rats. Eur J Pharmacol. (2004) 504:185–9. 10.1016/j.ejphar.2004.09.054 15541420

[14] Tsai CH Lin FM Yang YC Lee MT Cha TL Wu GJ. Herbal extract of wedelia chinensis attenuates androgen receptor activity and orthotopic growth of prostate cancer in nude mice. Clin Cancer Res. (2009) 15:5435–44. 10.1158/1078-0432.CCR-09-0298 19690196

[15] Yan L Meng QW Kim IH. The effect of an herb extract mixture on growth performance, nutrient digestibility, blood characteristics and fecal noxious gas content in growing pigs. Livest Sci. (2011) 141:143–7. 10.1016/j.livsci.2011.05.011

[16] Yan L Meng QW Kim IH. The effects of dietary houttuynia cordata and taraxacum officinale extract powder on growth performance, nutrient digestibility, blood characteristics and meat quality in finishing pigs. Livest Sci. (2011) 141:188–93. 10.1016/j.livsci.2011.05.017

[17] Huang CW Lee TT Shih YC Yu B. Effects of dietary supplementation of chinese medicinal herbs on polymorphonuclear neutrophil immune activity and small intestinal morphology in weanling pigs. J Anim Physiol An N. (2012) 96:285–94. 10.1111/j.1439-0396.2011.01151.x 21535231

[18] Liu WC Pi SH Kim IH. Effects of *Scutellaria baicalensis* and *Lonicera japonicaextract* mixture supplementation on growth performance, nutrient digestibility, blood profiles and meat quality in finishing pigs. Ital J Anim Sci. (2016) 15:446–52. 10.1080/1828051X.2016.1202736

[19] Costa LB Luciano FB Miyada VS Gois FD. Herbal extracts and organic acids as natural feed additives in pig diets. S Afr J Anim Sci. (2013) 43:181–93. 10.4314/sajas.v43i2.9

[20] Oetting LL Utiyama CE Giani PA Ruiz UDS Miyada VS. Effects of herbal extracts and antimicrobials on apparent digestibility, performance, organs morphometry and intestinal histology of weanling pigs. Rev Bras Zootecn. (2006) 35:2013–7. 10.1590/S1516-35982006000700019

[21] Ewa H Małgorzata S Eugeniusz RG. Effect of dietary inclusion of a herbal extract mixture and different oils on pig performance and meat quality. Meat Sci. (2015) 108:61–6. 10.1016/j.meatsci.2015.05.020 26047978

[22] Chinese Pharmacopeia Commission. Pharmacopoeia of the People's Republic of China (I Volumes). Peking: China Medical Science and Technology Press (2010).

[23] Frankic T Voljč M Salobir J Rezar V. Use of herbs and spices and their extracts in animal nutrition. Acta Agric Slov. (2009) 94:95–102. http://aas.bf.uni-lj.si/zootehnika/94-2009/PDF/94-2009-2-95-102.pdf

[24] Peeters E Driessen B Steegmans R Henot D Geers R. Effect of supplemental tryptophan, vitamin e, and a herbal product on responses by pigs to vibration. J Anim Sci. (2004) 82:2410–20. 10.2527/2004.8282410x 15318742

[25] Szewczyk A Hanczakowska E Swiatkiewicz M. The effect of nettle (*Urtica Dioica*) extract on fattening performance and fatty acid profile in the meat and serum lipids of pigs. J Anim Feed Sci. (2006) 15:81–84. 10.22358/jafs/70148/2006

[26] Wang Y Chen YJ Cho JH Yoo JS Wang Q Huang Y. The effects of dietary herbs and coral mineral complex on growth performance, nutrient digestibility, blood characteristics and meat quality in finishing pigs. J Anim Feed Sci. (2007) 16:395–8. 10.22358/jafs/66796/2007

[27] Yan L Meng QW Kim IH. Effect of an herb extract mixture on growth performance, nutrient digestibility, blood characteristics, and fecal microbial shedding in weanling pigs. Livest Sci. (2012) 145:189–95. 10.1016/j.livsci.2012.02.001

[28] Namkung H Li M Gong J Yu H Cottrill M Lange CD. Impact of feeding blends of organic acids and herbal extracts on growth performance, gut microbiota and digestive function in newly weaned pigs. Can J Anim Sci. (2004) 84:697–704. 10.4141/A04-005

[29] Liu WC Yun HM Pi SH Kim IH. Supplementing lactation diets with herbal extract mixture during summer improves the performance of sows and nursing piglets. Ann Anim Sci. (2017) 17:835–47. 10.1515/aoas-2016-0084

[30] Strathe AV Bruun TS Hansen CF. Sows with high milk production had both a high feed intake and high body mobilization. Animal. (2017) 11:1913–21. 10.1017/S1751731117000155 28196552

[31] Oliviero C Kokkonen T Heinonen M Sankari S Peltoniemi O. Feeding sows with high fibre diet around farrowing and early lactation: impact on intestinal activity, energy balance related parameters and litter performance. Res Vet Sci. (2009) 86:314–9. 10.1016/j.rvsc.2008.07.007 18725160

[32] Ilsley S Miller H Greathead H Kamel C. Herbal sow diets boost preweaning growth. Feed Mix. (2002) 10:24–5. 10.1017/S1752756200006797

[33] Zhong MD Wu D Lin Y Fang ZF. Phytogenic feed additive for sows: effects on sow feed intake, serum metabolite concentrations, IgG level, lysozyme activity and milk quality. J Agr Sci Tech-iran. (2011) 1:802–10. http://qikan.cqvip.com/Qikan/Article/Detail?id=40018923

[34] Watson DL. Immunological functions of the mammary gland and its secretion–comparative review. Aust J Biol Sci. (1980) 33:403–22. 10.1071/BI9800403 7004419

[35] Redoy MRA Shuvo AAS Cheng L Al-Mamun M. Effect of herbal supplementation on growth, immunity, rumen histology, serum antioxidants and meat quality of sheep. Animal. (2020) 14:2433–41. 10.1017/S1751731120001196 32498740

[36] Shokoh P Anousheh ZK Hamid RBR Hadi N Ahmad FI Safian S. Antioxidant, antimicrobial and antiviral properties of herbal materials. Antioxidants. (2020) 9:1309–45. 10.3390/antiox9121309 33371338 PMC7767362

[37] Yan SQ Wang YR Liu PP Chen A Chen MY Yao D. Baicalin attenuates hypoxia-induced pulmonary arterial hypertension to improve hypoxic cor pulmonale by reducing the activity of the p38 MAPK signaling pathway and MMP-9. Evid Based Complement Alternat Med. (2016) 2546402:1–9. 10.1155/2016/2546402 27688788 PMC5023842

[38] Meng S Cao XJM Feng Q Peng JH Hu YY. Roles of chlorogenic acid on regulating glucose and lipids metabolism: a review. Evid Based Complement Alternat Med. (2013) 2013:801457. 10.1155/2013/801457 24062792 PMC3766985

[39] Ruan Z Yang YH Zhou Y Wen YM Ding S Liu G. Metabolomic analysis of amino acid and energy metabolism in rats supplemented with chlorogenic acid. Amino Acids. (2014) 46:2219–29. 10.1007/s00726-014-1762-7 24927697 PMC5013734

[40] Myer RO Johnson DD Knauft DA Gorbet DW Brendemuhl JH Walker WR. Effect of feeding high-oleic-acid peanuts to growing-finishing swine on resulting carcass fatty acid profile and on carcass and meat quality characteristics. J Anim Sci. (1992) 70:3734–41. 10.2527/1992.70123734x 1474012

[41] Delcy V Rodriguez DS Hadley M. Chlorogenic acid modifies plasma and liver concentrations of: cholesterol, triacylglycerol, and minerals in (*fa/fa*) Zucker rats. J Nutr Biochem. (2002) 13:717–26. 10.1016/S0955-2863(02)00231-0 12550056

[42] Cho AS Jeon SM Kim MJ Yeo J Seo KI Choi MS. Chlorogenic acid exhibits anti-obesity property and improves lipid metabolism in high-fat diet-induced-obese mice. Food Chem Toxicol. (2010) 48:937–43. 10.1016/j.fct.2010.01.003 20064576

[43] Yotsumoto H Yanagita T Yamamoto K Ogaw Y Cha J Mori Y. Inhibitory effects of Oren-gedoku-to and its components on cholesteryl ester synthesis in cultured human hepatocyte GepG2 cells: Evidence from the cultured HepG2 cells and *in vitro* assay of ACAT. Planta Med. (1997) 63:141–5. 10.1055/s-2006-957631 9140228

[44] Hanczakowska E Witkiewicz M Grela ER. Effect of dietary supplement of herbal extract from hop (*Humulus lupulus*) on pig performance and meat quality. Czech J Anim Sci. (2017) 62:287–95. 10.17221/49/2016-CJAS

[45] Fermont L Gozzelino MT Linard A. Response of plasma lipids to dietary cholesterol and wine polyphenols in rats fed polyunsaturated fat diets. Lipids. (2000) 35:991–9. 10.1007/s11745-000-0610-2 11026620

[46] Huo B He J Shen XY. Effects of selenium-deprived habitat on the immune index and antioxidant capacity of *Przewalski's Gazelle*. Biol Trace Elem Res. (2020) 198:149–56. 10.1007/s12011-020-02070-6 32040847

[47] Chen WP Tang JL Bao JP Hu PF Shi ZL Wu LD. Anti-arthritic effects of chlorogenic acid in interleukin-1β-induced rabbit chondrocytes and a rabbit osteoarthritis model. Int Immunopharmacol. (2011) 11:23–8. 10.1016/j.intimp.2010.09.021 20951230

[48] Kim HR Lee DM Lee SH Seong AR Gin DW Hwang JA. Chlorogenic acid suppresses pulmonary eosinophilia, ige production, and th2-type cytokine production in an ovalbumin-induced allergic asthma: activation of stat-6 and jnk is inhibited by chlorogenic acid. Int Immunopharmacol. (2010) 10:1242–8. 10.1016/j.intimp.2010.07.005 20637839

[49] Liu LL Gong LK Wang H Xiao Y Wu XF Zhang YH. Baicalin inhibits macrophage activation by lipopolysaccharide and protects mice from endotoxin shock. Biochem Pharmacol. (2008) 75:914–22. 10.1016/j.bcp.2007.10.009 18191816

[50] Park SW Lee CH Kim YS Kang SS Jeon SJ Son KH. Protective effect of baicalin against carbon tetrachloride-induced acute hepatic injury in mice. J Pharmacol Sci. (2008) 106:136–43. 10.1254/jphs.FP0071392 18187930

[51] Kielland C Rootwelt V Reksen O Framstad T. The association between immunoglobulin G in sow colostrum and piglet plasma. J Anim Sci. (2015) 93:4453–62. 10.2527/jas.2014-8713 26440345

[52] Wang Q Kim HJ Cho JH Chen YJ Yoo JS Wang Y. Effects of phytogenic substances on growth performance, digestibility of nutrients, faecal noxious gas content, blood and milk characteristics and reproduction in sows and litter performance. J Anim Feed Sci. (2008) 17:362–78. 10.22358/jafs/66469/2008

[53] Huo B Wu T Song CJ Shen XY. Studies of selenium defciency in the Wumeng semi-fine wool sheep. Biol Trace Elem Res. (2020) 194:152–8. 10.1007/s12011-019-01751-1 31147978

[54] Liu YH Huo B Chen ZP Wang K Huang LJ Che LQ. Effects of organic chromium yeast on performance, meat quality, and serum parameters of grow-finish pigs. Biol Trace Elem Res. (2022) 200:1–9. 10.1007/s12011-022-03237-z 35524021

[55] Kimura Y Kubo M Tani T Arichi S Okuda H. Studies on *Scutellariae Radix*. IV Effect on lipid peroxidation in rat liver. Chem Pharm Bull. (1981) 29:2610–7. 10.1248/cpb.29.2610 7349281

[56] Su DY Li S Zhang W Wang J Wang JJ Lv MH. Structural elucidation of a polysaccharide from *Lonicera japonica* flowers, and its neuroprotective effect on cerebral ischemia-reperfusion injury in rat. Int J Biol Macromol. (2017) 99:350–7. 10.1016/j.ijbiomac.2017.02.096 28254571

[57] Wang ZL Wang S Kuang Y Hu ZM. A comprehensive review on phytochemistry, pharmacology, and flavonoid biosynthesis of *Scutellaria baicalensis*. Pharm Biol. (2018) 56:465–84. 10.1080/13880209.2018.1492620 31070530 PMC6292351

[58] Huang WH Lee AR Yang CH. Antioxidative and anti-Inflammatory activities of polyhydroxyflavonoids of *Scutellaria Baicalensis* GEORGI. Biosci Biotech Bioch. (2006) 70:2371–80. 10.1271/bbb.50698 17031041

[59] Choi CW Jung HA Kang SS Choi JS. Antioxidant constituents and a new triterpenoid glycoside from *Flos Lonicerae*. Arch Pharm Res. (2007) 30:1–7. 10.1007/BF02977770 17328234

[60] Hoving LL Soede NM Feitsma H Kemp B. Lactation weight loss in primiparous sows: consequences for embryo survival and progesterone and relations with metabolic profiles. Reprod Domest Anim. (2012) 47:1009–16. 10.1111/j.1439-0531.2012.02007.x 22420822

[61] Berchieri-Ronchi CB Kim SW Zhao Y Correa CK Yeum J Ferreira ALA. Oxidative stress status of highly prolific sows during gestation and lactation. Animal. (2011) 5:1774–9. 10.1017/S1751731111000772 22440418

